# Ollier Disease: A Case Series and Literature Review

**DOI:** 10.15388/Amed.2021.28.1.8

**Published:** 2021-02-19

**Authors:** Vėtra Markevičiūtė, Medeinė Šilenė Markevičiūtė, Mindaugas Stravinskas

**Affiliations:** Department of Orthopaedics and Traumatology, Lithuanian University of Health Sciences, Kauno klinikos, Kaunas, Lithuania; Faculty of Medicine, Vilnius University, Vilnius, Lithuania; Department of Orthopaedics and Traumatology, Lithuanian University of Health Sciences, Kauno klinikos, Kaunas, Lithuania

**Keywords:** Ollier disease, enchondroma, chondrosarcoma, echondromatosis

## Abstract

**Summary. Background.:**

Ollier disease is the most common nonhereditary type of enchondromatosis. Enchondromas are common, usually benign intraosseous cartilaginous tumors that form near the growth plate cartilage predominantly unilaterally in the metaphyses and diaphyses of tubular bones. They usually affect the long bones of the hand, the humerus, and the tibia, followed by flat bones, such as the pelvis. The estimated prevalence of Ollier disease is 1 in 100,000 and while it is linked with somatic heterozygous mutations in IDH1 or IDH2 genes, exact etiology is unknown. The risk of malignant transformation towards chondrosarcoma is up to 30–35% and it is clinically suspected when pain and a rapid increase in the size of the lesions is seen.

**Case presentations.:**

We report two clinical cases of patients diagnosed with Ollier disease. In both cases transformation to chondrosarcoma was observed.

**Conclusions.:**

Ollier disease is a rare disorder, defined by the presence of multiple enchondromas and an asymmetric distribution of the cartilage lesions that can be extremely variable in terms of size, location, age, gender. Constant monitoring of patients is important due to the high risk of malignancy. Because the disease is very rare and the manifestations vary widely, each patient’s case must be evaluated, and the treatment strategy adopted individually.

## Abstract (lt)

**Ollierio liga: klinikinių atvejų serija ir literatūros apžvalga:**

**Santrauka. Apžvalga.**

Ollierio liga yra dažniausias nepaveldimos enchondromatozės tipas. Enchondromos yra dažni gerybiniai kremzliniai navikai, susidarantys šalia augimo plokštelės kremzlės, vienoje pusėje, vamzdinių kaulų metafizėse ir diafizėse. Jie dažniausiai paveikia ilguosius rankos kaulus – žastikaulį, taip pat blauzdikaulį, rečiau plokščiuosius kaulus, pavyzdžiui, dubenį. Apskaičiuotas Ollierio ligos paplitimas yra 1 iš 100 000 ir, nors ji yra siejama su somatinėmis heterozigotinėmis IDH1 arba IDH2 genų mutacijomis, tiksli ligos etiologija nežinoma. Piktybinės transformacijos į chondrosarkomą rizika yra iki 30–35 % ir ji kliniškai įtariama, kai matomas greitas destrukcijos didėjimas ir yra skausmas.

**Klinikiniai atvejai.**

Pristatomi dviejų pacientų, kuriems diagnozuota Ollierio liga, klinikiniai atvejai. Abiem atvejais pacientams buvo nustatyta transformacija į chondrosarkomą.

**Išvados.**

Ollierio liga yra reta, jai būdingos daugybinės enchondromos ir asimetriškas kremzlės destrukcijų pasiskirstymas, kuris gali būti labai įvairus pagal dydį, vietą, paciento amžių ir lytį. Nuolat stebėti pacientus yra svarbu dėl didelės enchondromų supiktybėjimo rizikos. Kadangi liga yra labai reta ir jos raiška labai skirtinga, reikia įvertinti kiekvieno paciento atvejį ir nustatyti individualią gydymo strategiją.

**Raktažodžiai**: Ollierio liga, enchondroma, chondrosarkoma, enchondromatozė.

## Introduction

Enchondromas are common intraosseous cartilaginous tumors that form near the growth plate cartilage and are usually benign [1]. They predominantly occur unilaterally in the metaphyses and diaphyses of tubular bones. They usually affect the long bones of the hand, the humerus, and the tibia, followed by flat bones, such as the pelvis [2]. When multiple enchondromas are present, the condition is called enchondromatosis [1]. Most common nonhereditary type of echondromatosis is Ollier disease [3]. The etiology of Ollier disease is unknown [4], but it is linked with somatic heterozygous mutations in IDH1 or IDH2 genes [3,4]. The estimated prevalence of Ollier disease is 1 in 100,000 [1, 2], while the risk of malignant transformation towards chondrosarcoma is up to 30–35%. Malignancy is clinically suspected when pain and a rapid increase in the size of the lesions is seen [4, 5]. Here we present two cases of Ollier disease from the orthopedic traumatology clinic in Hospital of Lithuanian University of Health Sciences (HLUHS).

## Case Reports

### CASE 1

**Fig. 1.**
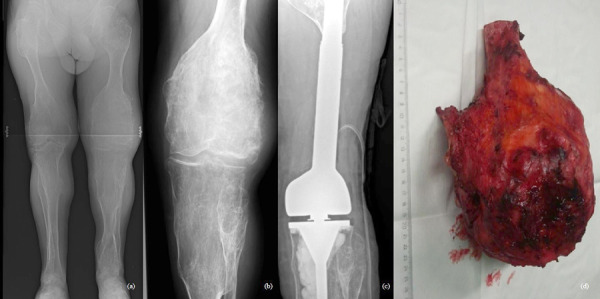
a-b) On December, 2015, radiographs showed multiple destructive lesions in the legs; c) on March, 2016, resection of the left bottom part of femur and arthroplasty with megaprosthesis; d) resected tumor – distal part of femur. Chondrosarcoma G3

**Fig. 2.**
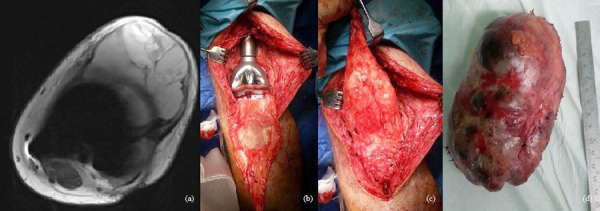
a) Chondrosarcoma G3, local reoccurrence in patella after 1year (MRI); b-c) 2017, we performed resection and transplantation of patella from a donor (operating room view); d) resected tumor – patella. Chondrosarcoma G3.

**Fig. 3.**
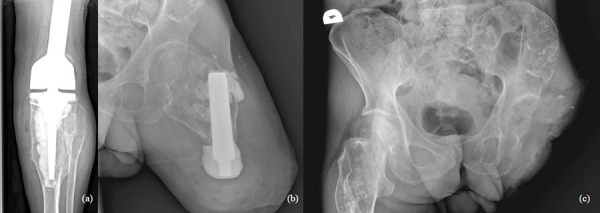
a) December 2018, local reoccurrence in proximal tibiae. Curettage of the tumor was performed; b) 2020, amputation in the middle third of the thigh; c) 2020, exarticulation through the hip joint.

47-year-old man treated for 6 years in HLUHS for Ollier disease and chondrosarcoma. The patient underwent several surgeries in childhood due to leg deformities, different leg lengths – surgeries were performed with Ilizarov apparatus. In 2015 patient was referred to HLUHS outpatient clinic for long-term tiring pain in the left leg, difficulty walking – symptoms were intense and present for 5 years. Deformations were present in both legs. It was difficult to determine malignant changes due to severe anatomical changes. No radiographs were performed in 10 years. Arthritic changes were identified. Due to continuous pain, patient was consulted again after 8 months. Radiological examinations showed multiple zones of destruction, sclerosis in the distal part of the femur and an increase in bone density in the proximal part of the tibia. Progression of destructions was also seen. A biopsy was performed in January 2016, during which he was diagnosed with acute malignant chondrosarcoma G3. The production of a specialized oncological knee prosthesis, which was planned to be used during reconstruction after radical treatment, took 3 months. The patient was examined for the spread of the disease since chondrosarcoma metastases are most likely observed in the lungs [6]. During this time the disease progression was observed – lung metastases. In March 2016, tumor intraarticular resection and left knee replacement surgery were performed (Fig.1), partial resection of patella was performed due to observed changes, followed by adjuvant chemotherapy VIP (etoposide, ifosfamide, and cisplatin) according to the VIP protocol [7], and the treatment tactic was selected according to the ESMO Clinical Practice Guidelines for diagnosis, treatment, and follow-up [8]. In January 2017, relapse of the patella was observed. Patient refused amputation because metastases were diagnosed in the lungs. Due to observed changes patella resection was performed, followed by a reconstruction with donor patella and transplantation (Fig. 2). After surgery, VIP chemotherapy was continued till December 2017. In 2 years (2018–2019) 3 excisions of new malignant nodules in left tibia. The patient walked at bearing full weight, worked, knee flexion of 60 degrees was satisfactory for the patient. In 2020 amputation in the middle third of the thigh was performed because of possible pathological fracture, later 1 exarticulation through the hip joint was completed (Fig. 3). In total, patient had 7 surgeries. Currently it is 5 years of LWD (life with disease).

### CASE 2

**Fig. 4.**
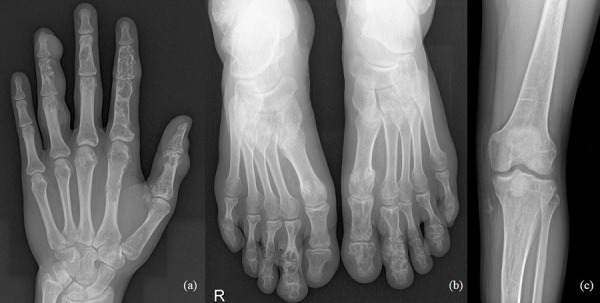
a) Multiple enchondromas in the left hand, about 15 enchondroma foci in all phalanges in fingers I-IV and in metacarpal bones I-IV; b) multiple enchondromas in the foot, about 10 enchondroma foci in toes I-V and metatarsals; c) enchondroma in the proximal part of left tibia.

**Fig. 5.**
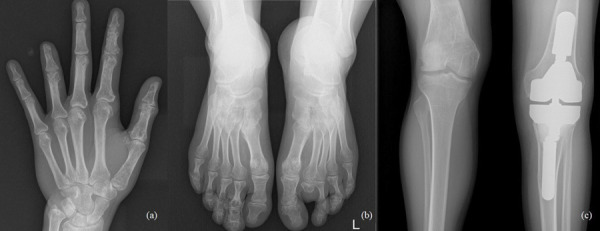
a) 2020, after amputation of the distal phalanx of IV finger was performed, 13 left hand enchondroma curettages were performed, most of which were performed with cancellous bone grafting; b) 2020, after II toe amputation and 3 curettages with cancellous bone grafting; c) 2020, resection of the tumor recurrence and arthroplasty with a special prosthesis.

The 33-year-old man is actively treated and followed-up in HLUHS for Ollier disease for 3 years. Patient was followed-up by a pediatric orthopedic surgeon during his childhood. Due to adequate well-being patient did not seek medical attention for years. In 2017, he consulted an orthopedic oncologist complaining of formations and pain in the left hand. Performed studies showed these changes (Fig. 4): multiple enchondromas in the left hand, about 15 enchondroma foci in all phalanges in fingers I-IV and in metacarpal bones I-IV, about 10 enchondroma foci in toes I-V and metatarsals in the left foot, enchondroma in the proximal part of left tibia. The patient is followed-up every 3 months. In 2018 growth of the distal phalanx of the IV finger was observed – surgery was performed due to suspected malignancy, amputation of the distal phalanx of IV finger was performed (chondrosarcoma G2 was confirmed), curettage of the II finger enchondroma with cancellous bone grafting. The patient was hospitalized 4 more times in 1 year – 12 left hand enchondroma curettages were performed, most of which were performed with cancellous bone grafting. The left foot was operated 4 times – II toe amputation and 3 curettages with cancellous bone grafting, the enchondroma curettage with cancellous bone grafting of tibia’s proximal part. In 2 years (2018–2019), the patient was hospitalized 7 times, during which a total of 21 surgeries in different locations of bone were performed to protect from pathological fractures (Fig. 5). In February 2019, he underwent surgery for a reoccurrence in the proximal part of the left tibia – curettage with cancellous bone grafting and cementoplasties. Histology showed chondrosarcoma G2. Full body CT was done followed by a PET scan. Patient was followed-up. In studies performed in October 2019, recurrence of the disease was observed, and a special prosthesis was ordered. Extensive tumor resection according to ESMO guidelines [9] and left knee replacement surgery were performed. At present, there is no data on relapse, and the patient is monitored by orthopedic traumatologists and oncologists every 3 months according to the ESMO Clinical Practice Guidelines for diagnosis, treatment, and follow-up [9]. The patient is walking at bearing full weight, worked, knee flexion is up to 80 degrees.

## Discussion

The enchondromas, while not apparent at birth, are typically painless and can result in short stature and limb length discrepancy [10], as reported in the first case, patient was treated for leg shortening as a child. Enchondromas, as mentioned in our second case, are usually located in the little finger (29–65%) and proximal phalange (53–60%) [11,12]. Enchondromatosis or Ollier disease is defined by the presence of multiple enchondromas and an asymmetric distribution of the cartilage lesions that can be extremely variable in terms of size, location, age of onset, quantity, and requirement for surgery [13]. Enchondromas occur equally in both sexes and are most discovered between 20 and 40 years of age. However, Ollier disease is seen twice as often in men than in women and tends to present before 10 years old, but it can also be diagnosed in adolescence and adulthood [6, 13]. The usual clinical manifestation of Ollier disease includes painless asymptomatic palpable bony masses on the fingers, toes, and metacarpals, with a unilateral predominance [14]. The masses increase in size as the child is growing and cause asymmetrical shortening of a limb and deformities like genu valgum and genu varum, last one being the most common [14]. Theory of Ollier disease is that the growing end of bone in which the normal ossification of cartilage fails to take place, so that, as the bone increases in length, there remain the diaphyses areas of cartilage which do not ossify. The process is not primarily that of tumor formation [15], however transformation to chondrosarcoma, as reported in both clinical cases, is often observed. Patients should be followed-up regularly due to the high risk of malignancy, which based on literature is 30–35% [4,5].

Ollier disease is a rare disease that can be diagnosed relatively easily solely on clinical grounds and considering a few simple ancillary tests, namely radiographs [16]. The disease has specific radiographic features representing unilateral involvement with presence of multiple lytic lesions in the center of tubular bones of hand, foot, and long bones, particularly in metaphyseal regions. Sometimes there is symmetrical involvement but still with unilateral predominance [17]. Plain radio-graphs frequently reveal multiple lytic lesions in the affected area, with significant erosion, deep endosteal scalloping, usually greater than two-thirds of cortical thickness and no significant periosteal reaction [14]. During the course of the disease pathologic fractures are seen in some patients due to the cortical thinning [17]. Based on data reviews pathologic fractures are observed in 40%-60% of patients at initial presentation of the disease [9]. 

Computed tomography (CT) is superior to radiography in detecting matrix mineralization, calcification pattern, lobulated lesion margins, and degree and extent of endosteal scalloping [6]. Enchondromas appear as hypo- or hyperdense lobulated and well-defined mass with mild contrast enhancement on CT scan [18]. It is highly indicated in evaluation of areas where radiography is impossible, such as the pelvis [19]. CT is particularly useful in evaluating the size and presence of soft tissue components, which would favor a diagnosis of chondrosarcoma [6,19]. Scintigraphy, ultrasound, and other additional tests have limited application in the diagnosis of enchondromas and chondromas but may be requested in cases of pathological fracture when lesion characterization is necessary prior to treatment [6, 13, 19]. Both CT and MRI are irreplaceable additional tools for bone lesion assessment. MRI is especially useful in evaluating intraosseous and soft tissue involvement [18]. 

Murphy et al. analysis names criteria that allows distinction of chondrosarcoma and enchondroma in at least 90% of cases. According to their study 100% of enchondromas and 94% of chondrosarcomas can be identified by mineralized matrix in CT. In addition, multiple clinical and imaging factors – particularly pain related to the lesion, depth of scalloping greater than two thirds of cortical thickness, cortical destruction, and soft-tissue mass (CT or MRI). Periosteal reaction (at radiography), and uptake greater than the anterior iliac crest at bone scintigraphy strongly suggest the diagnosis of chondrosarcoma [20]. In a newer clinical study Eugenio M. Ferrer-Santacreu et al. employed an aggressiveness score for doubtful cases to complement clinical and radiological data, but in no way it can be considered as an absolute indicator of malignancy. Total aggressiveness score was counted by adding 1 point for each following feature from 3 categories (Fig. 6). Their analysis established that those patients with a total aggressiveness score of 5 or more had a risk of having low grade chondrosarcoma higher than 50%. Authors also highlight the main clinical and radiological features to help differentiate between solitary enchondroma and low-grade chondrosarcoma [21]. Julia Crim et al. argue that both radiographs and MRI have limitations in the evaluation of low-grade cartilage lesions. MRI has an increased rate of both true-positive and false-positive diagnosis compared to radiographs [22]. We believe that due to the high risk of malignancy, constant monitoring with an experienced radiologist is important for early changes to be identified. Only after chondrosarcoma is suspected, specialized evaluation is required.

**Fig. 6.** Aggressiveness score employed in Eugenio M. Ferrer-Santacreu et al. study.

Aggressiveness categories	Features (1 point per each of the following features)
Clinical aggressiveness (CA)	Presence of inflammatory pain
	Presence of pain with palpation
Radiological aggressiveness (RA)	Size bigger than 5 cm
	Metaphyseal location
	Loss of calcification (calcification lysis) over time
	Cortical involvement in CT or MRI
	Presence of a soft tissue mass in CT or MRI
Metabolic aggressiveness (MA)	Presence of Tc99 uptake in bone scan
	Uptake equal to or higher than anterosuperior iliac crest (ASIC)
Total aggressiveness (TA)	=CA + RA + MA

**Fig. 7.** Compared features of solitary enchondroma and low grade chondrosarcoma.

Features	Solitary enchondroma	Low grade chondrosarcoma
Clinical	1. Younger patients	1. Patients > 25 years
	2. Pain is rare	2. Inflammatory pain
	3. Typical in appendicular skeleton	3. Axial skeleton
	4. In general, <5 cm	4. Bigger size
Radiological	1. Intramedullary	1. Intramedullary
	2. No periosteal reaction	2. Periosteal reaction and microfractures
	3. No endosteal scalloping	3. Endosteal scalloping
	4. No changes over time	4. Loss of calcification. Increasing size
	5. No soft tissue mass	5. Soft tissue mass in some cases

Prognosis is difficult to be assessed because of wide clinical variability in the presentation of the disease [26]. Poor prognosis has been identified with early age of onset, malignant transformation, and gross asymmetrical distribution [10]. Patient with numerous lesions may have a better prognosis than patients with localized cartilaginous changes, which may induce major shortening of a lower extremity and thus limb asymmetry, especially if already present in a very young age. Similarly, early development of enchondromas in phalanges may lead to major finger deformities [1]. Chondrosarcomas are the most common malignancy arising from Ollier disease and are present in approximately 25% of patients by age 40 years [6]. Usually, chondrosarcomas grow slowly and rarely metastasize, and they have an excellent prognosis after adequate surgery. However, a minority of patients present or recur with metastatic disease, and up to 13% of recurrent chondrosarcomas are of a higher grade than the original neoplasm [23].

Craig C. Akoh et al. recommend surveillance with plain radiographic follow-up for stable enchondromas every 3–6 months for the first year and then annually for at least three years of total follow-up [27]. Musculoskeletal Tumor Society (MSTS) published in 2018 guidelines for surveillance of newly identified bone lesions. For asymptomatic stable lesions, they recommended serial radiographs and review by a fellowship-trained musculoskeletal radiologist every 3–6 months for 2 years. Observed progression of radiological findings or new symptoms warrants immediate referral to an orthopedic oncologist [28]. National Comprehensive Cancer Network (NCCN) recommended a follow-up consisting of a physical exam: radiographs of primary site and/or cross-sectional imaging MRI or CT (both with contract) and chest imaging. For low-grade bone sarcomas follow up is indicated every 6 months for 2 years, and then once a year. For surveillance of high-grade chondrosarcomas previously mentioned tests should be performed and additional chest CT (with or without contrast) every 3–6 months for 5 years, then yearly for minimum of 10 years, based on physician’s concern for risk of recurrence. Functional assessment also should be performed at every visit [29].

## Conclusion

Ollier disease is a rare disorder, defined by the presence of multiple enchondromas and an asymmetric distribution of the cartilage lesions that can be extremely variable in terms of size, location, age, gender. Due to the high risk of malignancy, constant monitoring with an experienced radiologist every 3–6 months is important for early changes to be identified. Only after chondrosarcoma is suspected, specialized evaluation is required. Because the disease is rare and the manifestations varies widely, each patient’s case must be evaluated, and the treatment strategy adopted individually in a specialized orthopedic oncology center. 
